# Ophiopogonin D′, a Natural Product From *Radix Ophiopogonis*, Induces *in Vitro* and *in Vivo* RIPK1-Dependent and Caspase-Independent Apoptotic Death in Androgen-Independent Human Prostate Cancer Cells

**DOI:** 10.3389/fphar.2018.00432

**Published:** 2018-04-30

**Authors:** Zongliang Lu, He Wang, Mingxing Zhu, Wei Song, Jiajia Wang, Changpeng Wu, Ya Kong, Jing Guo, Na Li, Jie Liu, Yanwu Li, Hongxia Xu

**Affiliations:** ^1^Department of Nutrition, Daping Hospital and Research Institute of Surgery, Third Military Medical University, Chongqing, China; ^2^Department of Clinical Nutrition, Yubei District People’s Hospital, Chongqing, China; ^3^Pharmacy College, Chongqing Medical University, Chongqing, China

**Keywords:** Ophiopogonin D′, RIPK1, Bim, apoptosis, prostate cancer

## Abstract

**Objective:** The purpose of this study was to evaluate the anticancer effects of Ophiopogonin D′ (OPD′, a natural product extracted from a traditional Chinese medicine (*Radix Ophiopogonis*) against androgen-independent prostate cancer cells and to explore the underlying molecular mechanism(s) of action.

**Methods:** The CCK-8 assay was used to assess the viability of prostate cancer cells. The cell morphology was examined by an ultrastructural analysis via transmission electron microscopy. Cells in apoptosis (early and late stages) were detected using an Annexin V-FITC/propidium iodide kit with a FACSCaliber flow cytometer. JC-1, a cationic lipophilic probe, was employed to measure the mitochondrial membrane potential (MMP) of PC3 cells. Changes in the protein expression of RIPK1, C-RIPK1, caspase 8, cleaved-caspase 8, Bim, Bid, caspase 10, and cleaved-caspase 10 were evaluated by Western blotting. The mRNA expression of Bim was examined by quantitative real-time reverse transcription polymerase chain reaction. Z-VAD-FMK (a caspase inhibitor) and necrostatin-1 (a specific inhibitor of RIPK1) were utilized to determine whether the cell death was mediated by RIPK1 or caspases. PC3 and DU145 xenograft models in BALB/c nude mice were used to evaluate the anticancer activity of OPD′ *in vivo*.

**Results:** OPD′ was shown to exert potent anti-tumor activity against PC3 cells. It induced apoptosis via a RIPK1-related pathway, increased the protein expression levels of RIPK1 and Bim, and decreased the levels of cleaved-RIPK1, caspase 8, cleaved-caspase 8, Bid, caspase 10, and cleaved-caspase 10. OPD′ also increased the mRNA expression of Bim. The protein expression of Bim was decreased when cells were pre-treated with necrostatin-1. Treatment with OPD′ inhibited the growth of PC3 and DU145 xenograft tumors in BALB/c nude mice.

**Conclusion:** OPD′ significantly inhibited the *in vitro* and *in vivo* growth of prostate cells via RIPK1, suggesting that OPD′ may be developed as a potential anti-prostate cancer agent.

## Introduction

Prostate cancer is the most common male urogenital malignancy in developed countries (Siegel et al., 2018). Early stages of the disease can be managed with active surveillance, radical prostatectomy or radiation therapy. However, no curative treatment exists for advanced disease [i.e., castration-resistant prostate cancer (CRPC)], which eventually develops in approximately 30% of patients with prostate cancer (Climent et al., 2015). Chemotherapy is usually). recommended for patients with CRPC based on the National Comprehensive Cancer Network (NCCN) Guidelines (Mohler et al., 2016), but some studies have shown that conventional chemotherapy is ineffective against CRPC (Zhou et al., 2016; Li et al., 2018). Therefore, there is an urgent need to discover new drugs and targets for the treatment of prostate cancer, especially advanced disease.

Apoptosis and necrosis have been linked to the development and progression of various cancers, and are therapeutic targets for various cancers (Thompson et al., 1995; Feng et al., 2016; Renner et al., 2017). Receptor interacting protein 1 (RIP1) kinase is a crucial regulator of cell survival and cell death, and is emerging as an important regulator of the cell fate in response to cellular stress (Luan et al., 2015). When the activation of caspase 8 is limited, deubiquitinated receptor interacting serine/threonine-protein kinase 1 (RIPK1) recruits RIPK3, leading to programmed necrosis (Bertrand et al., 2008; Wang et al., 2008). In addition, RIPK1 affects the mitochondrial membrane integrity, resulting in the release of proteins from the mitochondrial inter-membrane space via c-Jun N-terminal kinase (JNK) (Festjens et al., 2007). Sustained JNK activation is known to trigger apoptosis by regulating the activity of death-related genes such as Bcl-2-like protein 11 (Bim) (Xia et al., 2007). Several existing clinically-used drugs have been shown to function at least partially via RIPK1. For example, treatment with simvastatin and metformin induced G1-phase cell cycle arrest in (CRPC) cells in a RIPK1-dependent manner (Babcook et al., 2014). Sorafenib promoted the interaction of RIPK1 with p62 to induce necroptosis in DU145 prostate cancer cells (Kharaziha et al., 2015).

Natural products have provided an invaluable source of therapeutic agents, especially for anticancer drug discovery. Terpenoid saponins, including the triterpenoid saponins and diterpenoid saponins identified from many herbs, have been demonstrated to have activity against human cancer cells (Haridas et al., 2001; Mujoo et al., 2001; Liu et al., 2013; Cheng et al., 2014; Chun et al., 2014; Du et al., 2014; Zhou et al., 2015; Singh et al., 2017). We recently examined four triterpenoid saponins and one diterpenoid saponin for their antitumor effects against androgen-independent PC3 prostate cancer cells. These compounds included Ophiopogonin D′(OPD′) and Ophiopogonin D (OPD) from *Ophiopogon japonicus*, Liriopesides B (LB) from *Liriope spicata (Thunb.) Lour., Liriope muscari* baily saponins C (LSC) from *Liriope muscari (Decne.) Bailey*., and Darutoside (DS) (a diterpenoid saponin) from *Siegesbeckia orientalis L.* Our data showed that OPD′ exhibited potent growth inhibitory activity against PC3 cells, but the precise molecular mechanisms underlying the anti-cancer effects of this compound are still being determined. In this study, we investigated the anti-cancer effects and mechanism(s) of action of OPD′ using *in vitro* and *in vivo* prostate cancer models.

## Materials and Methods

### Test Compounds, Chemicals, and Reagents

**FIGURE 1:**
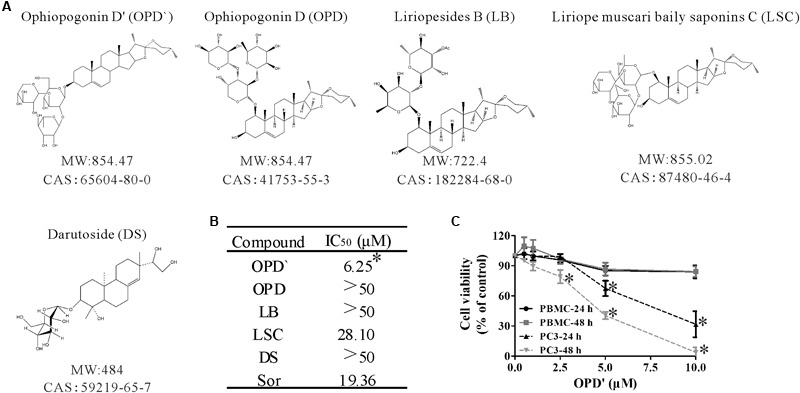
The chemical structures and anticancer activity of five compounds. **(A)** The chemical structures of the compounds. **(B)** The concentrations of the five compounds and one positive control (Sorafenib) that induced 50% growth inhibition (IC_50_) in PC3 cells after 24 h of exposure. *n* = 3 independent experiments. ^∗^*p* < 0.05 vs. OPD, LSC, LB, or DS. **(C)** After being treated with various concentrations of OPD′ for 24 or 48 h, the viability of PBMC or PC3 cells was checked using the CCK-8 assay. PBMC were isolated from whole blood obtained from seven healthy donors. *n* = 3 independent experiments. ^∗^*p* < 0.05 vs. 0 μM OPD′ treatment.

Four triterpenoid saponins (**Figure 1A**), OPD′, OPD, LSC, LB, and a diterpenoid saponin (DS), were evaluated for anti-cancer activity in human prostate cancer cells. All five compounds were purchased from Must Bio-Technology, Co., Ltd. (Chengdu, China). The structures of the five test compounds were confirmed based on their nuclear magnetic resonance (NMR) spectra (**Supplementary Data Sheet S4**). The purity of test compounds (all >96%; **Supplementary Data Sheet S3**) was determined by high-performance liquid chromatography (HPLC). Fetal bovine serum (FBS) was obtained from BIOIND (Biological Industries, Beit HaEmek, Israel). Sorafenib (positive control) was purchased from Selleck, Co., Ltd. (Shanghai, China). The anti-human RIPK1, anti-C-RIPK1, anti-caspase 8, anti-C-caspase 8, anti-Bim, anti-caspase 10, anti-C-caspase 10, and anti-Bid antibodies were purchased from Cell Signaling Technology, Inc. (Danvers, MA, United States). Necrostatin-1 (Nec-1) and Z-VAD-FMK were purchased from Selleckchem (Houston, TX, United States).

### Cell Lines and Cell Culture

Androgen-independent prostate cancer cell lines, PC3 (**Supplementary Data Sheet S1**) and DU145 (**Supplementary Data Sheet S2**), were obtained from the American Type Culture Collection (Manassas, VA, United States). The PC3 cells were grown in DMEM/Ham’s F12 medium supplemented with 10% FBS. The DU145 cells were cultured in RPMI 1640 medium supplemented with 10% FBS. Peripheral blood mononuclear cells (PBMC) were cultured in RPMI 1640 medium supplemented with 10% FBS, 2 mmol/L glutamine, and 0.1% gentamycin. Third-passage prostate cancer cells were used in all of the experiments.

### PBMC Separation

The PBMC were isolated by density centrifugation of whole blood obtained from healthy donors. In brief, an equal volume of 0.01 M phosphate-buffered saline (PBS) with 10 UI/ml heparin (Changshan Biochemical Pharmaceutical, Co. Ltd., Hebei, China) was added to whole blood, which was then mixed to obtain a cell suspension. Subsequently, 5 ml of the resulting whole blood cell suspension was added on the top of 5 ml 60% percoll layered liquid (GE Healthcare, Co., Beijing, China), and then centrifuged at 600 g/min for 30 min. The top liquid layer (plasma) was removed, and the cells (PBMC) in the boundary between the top and bottom layered liquids were harvested. After isolation, the PBMC were washed three times in PBS containing 2% FBS and 5 UI/ml heparin.

### Cell Survival Assay

The effects of the five terpenoid saponins on cell growth were determined using the CCK-8 assay. The cells were exposed to various concentrations (1, 2.5, 5, 10, 25, and 50 μM) of the five compounds and Sorafenib [a positive control compound (Kharaziha et al., 2015)]. The absorbance at 450 nm was then recorded using a TECAN Infinite M200 microplate reader (Seestraße, Switzerland). The cell survival rates (%) were calculated based on the ratio of the mean OD of compound-treated wells divided by that of DMSO-treated control wells.

### Apoptosis Assay

Apoptosis was assessed using our lab’s previously-reported protocol (Lu et al., 2016) with an Annexin V-FITC/PI apoptosis detection kit (BestBio, Shanghai, China). The cells (2.0 × 10^5^/well) were grown in 6-well plates that were exposed to OPD′(2.5 or 5.0 μM) or Sorafenib (5.0 or 10.0 μM) for 18 h, and then incubated with Annexin V-FITC/propidium iodide (PI) for 15 min prior to the analysis using a FACSCaliber flow cytometer (BD Biosciences, San Jose, CA, United States). Cells in early apoptosis were Annexin V-FITC-positive and PI-negative (FITC^+^/PI^-^), while cells that were dead or in late apoptosis were both Annexin V- FITC- and PI-positive (FITC^+^/PI^+^). Upon finding that OPD′ induced apoptosis, the PC3 cells were treated with or without Nec-1 or Z-VAD-FMK to determine whether the apoptosis was RIPK1- or caspase-mediated.

### Ultrastructural Study of Apoptosis

A morphological observation of apoptotic cells was performed using transmission electron microscopy (TEM). The PC3 cells (1 × 10^6^) cultured in 25 mm^2^ culture flasks were treated with various concentrations of OPD′ for 6 h. The cells were then washed with PBS, fixed with1% osmium tetroxide and then observed by TEM (TECNAI 10, FEI Company, Holland).

### Measurement of the Mitochondrial Membrane Potential

A fluorescent, lipophilic and cationic probe, JC-1 (Beyotime, Shanghai, China), was employed to determine the mitochondrial membrane potential (MMP) (Δψm) of the PC3 cells. Briefly, a total of 2.0 × 10^5^ cells/well grown in 6-well plates were cultured with various concentrations of OPD′ for 6 h. The cell suspensions were collected and washed twice with PBS, followed by incubation with JC-1 staining solution (5 μg/mL) for 20 min at 37°C. The cells were then rinsed twice with JC-1 staining buffer. The fluorescence intensity of both mitochondrial JC-1 monomers (λex 514 nm and λem 529 nm) and aggregates (λex 585 nm and λem 590 nm) were detected using a FACSCaliber flow cytometer (BD Biosciences, San Jose, CA, United States). The Δψm of PC3 cells in each group were calculated based on the green fluorescence ratio (i.e., monomers).

### RNA Extraction, Reverse Transcription-PCR, and Real-Time Quantitative PCR

Total RNA was extracted using the Trizol reagent from BioFlux (Hangzhou Bioer Technology, Co., Ltd., Hangzhou, China). A 1 μg aliquot of RNA from each sample was reverse transcribed. The primer sequences used for gene amplification were as follows: Bim forward, 5′-TCCCTACAGACAGAGCCACA-3′ and reverse, 5′-CTTCACCTCCGTGATTGCCT-3′; and β-actin forward, 5′-AGCCTCGCCTTTGCCGA-3′ and reverse, 5′-CTGGTGCCTGGGGCG-3′. Using an iQ5 machine (Bio-Rad, Hercules, CA, United States), a 25 μl reaction mixture was amplified using the following parameters: denaturation at 94°C for 2 min and 40 cycles of the amplification step (94°C for 10 s, 60°C for 15 s, and 72°C for 45 s). This was followed by a final extension at 94°C for 2 min, 72°C for 1 min, 95°C for 30 s, and 30°C for 1 min. All of the amplification reactions were analyzed by the comparative threshold cycle (Ct) method, and data were normalized to the level of β-actin mRNA, which served as an internal control.

### Western Blotting Analysis

Cells (1 × 10^6^) cultured in 25 mm^2^ culture flasks were exposed to various concentrations of OPD′ for 6 h. Whole cell lysates were obtained by cell lysis in ice-cold RIPA buffer and were subjected to SDS-PAGE, according to a previously reported procedure (Lu et al., 2016). PVDF membranes with the adherent proteins were incubated with the selected primary antibody overnight at 4°C with gentle shaking. The membrane was then incubated with a goat anti-mouse/rabbit IgG horseradish peroxidase-conjugated secondary antibody (Bio-Rad, Hercules, CA, United States). Conjugated proteins were detected by the Fusion FX5 Spectra instrument from Vilber Lourmat, Inc. (Marne-la-Vallée, France).

### Mouse Xenograft Models

The animal care and use were performed in accordance with our institutional guidelines for the use of laboratory animals. All animal study procedures were approved by the Animal Ethics Committee of the Third Military Medical University. Male athymic pathogen-free nude mice (BALB/c, nu/nu, 4–6 weeks old) were purchased from the Medical Experimental Animal Center of the Third Military Medical University [SCXK-(army)-2007-015]. To establish human prostate cancer PC3 and DU145 xenograft tumor models, cultured cells were harvested and re-suspended in serum-free F12 (PC3) or 1640 medium (DU145) containing Matrigel (20% v/v; BD Biosciences, Bedford, MA, United States), and then injected subcutaneously (5 × 10^6^ cells) into the left inguinal area of the mice. All animals were monitored for activity, general condition, body weight, and tumor growth. The tumor size was measured every third day using calipers, and the tumor volume (cm^3^) was calculated by the following formula: (a × b^2^)/2, where ‘a’ and ‘b’ represent the longer and shorter dimensions.

### *In Vivo* Chemotherapy of the Tumor-Bearing Mice

One week after tumor cell inoculation, mice bearing palpable tumors were randomly divided into control and treatment groups (8 mice/group). OPD′ was dissolved in the vehicle, PEG400:Saline:Ethanol (400:300:200, v/v/v), and administered (via i.p. injection) at doses of 2.5 or 5.0 mg/kg bodyweight 5 days a week for 24 days. The control group received vehicle only. The mice were sacrificed by cervical dislocation on Day 24, and the tumor tissues were removed and weighed.

### Statistical Analysis

The data for the different treatment groups are presented as the means ± standard error. A one-way ANOVA was used to determine the significance of the effects of the treatments on cell viability, apoptosis, mRNA expression, tumor weight and body weight (after tumor removal). Repeated measures analyses of the body weight and tumor size were used to determine the significance of the *in vivo* findings. The results were considered to be significant for values of *p* < 0.05.

## Results

### Terpenoid Saponins Inhibit Prostate Cancer Cell Growth

Three independent CCK-8 assays were used to assess the viability of PC3 cells following exposure to OPD′, OPD, LB, LSC, and DS. Sorafenib, a compound known to suppress tumor growth, was selected as a positive control compound (Beardsley et al., 2012). PC3 cells were treated with several different concentrations of these compounds for 24 h, then the survival of the cells was determined. As shown in **Figure 1B**, the IC_50_ values for OPD′ and LSC were 6.25 and 28.10 μM, respectively. In this system, the IC_50_ value for Sorafenib was 19.56 μM. The IC_50_ values for the other three compounds, OPD, LB, and DS, were all >50 μM. Based on these preliminary findings, we elected to focus on OPD′ in the subsequent experiments.

### OPD′ Does Not Decrease the Viability of PBMC

The proliferative potential of PBMC cells is relevant for cancer defense (Boltz et al., 1987). The antiproliferative effects of many natural products may not be specific for cancer cells, and natural products may thus interfere with the proliferation of PBMC (Jenny et al., 2011). We therefore also examined the effects of OPD′ on the viability of human PBMC. As shown in **Figure 1C**, treatment with 10.0 μM OPD′ resulted in a decrease in the viability of PBMC by about 15% after 24 or 48 h. These results suggest that the compound was not toxic to PBMC at the effective concentration.

### OPD′ Induces Apoptosis in PC3 Cells

**FIGURE 2:**
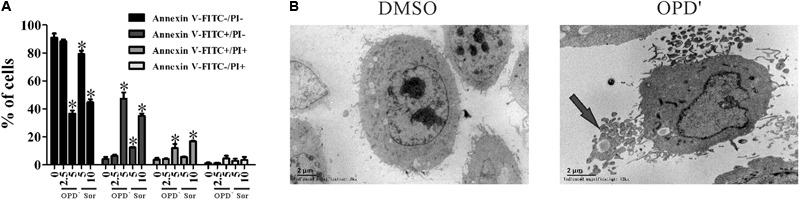
OPD′ induced PC3 cell apoptosis. **(A)** The Annexin V-FITC/PI assay was used check the apoptosis of PC3 cells treated with various concentrations of OPD′ or Sorafenib for 18 h. Data are presented as the means ± SD; Sor, Sorafenib. *n* = 3 independent experiments. ^∗^*p* < 0.05 vs. 0 μM. **(B)** Transmission electron microscopy (TEM) images of untreated and 5 μM OPD′-treated (6 h) PC3 cells. Arrow: apoptotic body.

The apoptosis analyses showed that treatment with 5.0 μM of OPD′, or 5.0 or 10.0 μM of Sorafenib, increased the FITC-positive (early apoptosis) and FITC/PI dual-positive (late apoptosis) areas for PC3 cells (**Figure 2A**). Conspicuous morphological changes indicating apoptosis were also observed in the TEM images (**Figure 2B**).

### The OPD′-Induced Apoptosis Is Mediated by RIPK1

**FIGURE 3:**
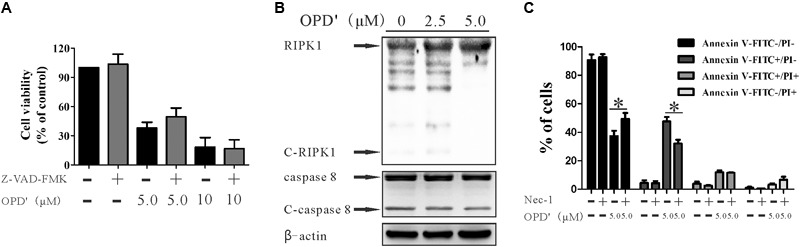
OPD′ induced early apoptosis through the RIPK1 pathway, not the caspase pathway. **(A)** After being pre-treated with 20 μM Z-VAD-FMK (a caspase inhibitor) for 2 h, the PC3 cells were exposed to various concentrations of OPD′ for 24 h, followed by the CCK-8 assay. Data are presented as the means ± SD; *n* = 3 independent experiments. **(B)** PC3 cells were exposed to various concentrations of OPD′ for 6 h, then target proteins (RIPK1, C-RIPK1, caspase-8, and C-caspase-8) were examined by Western blotting. *n* = 3 independent experiments; this panel is one of these replicates. **(C)** After pre-treatment with 10 μM Nec-1 (a specific inhibitor of the RIP1 kinase domain) for 2 h, PC3 cells were exposed to various concentrations of OPD′ for 18 h, followed by the Annexin V-FITC/PI assay. Data are presented as the means ± SD; *n* = 3 independent experiments. ^∗^*p* < 0.05 vs. treatment with 5 μM OPD′ alone.

The caspase pathway is one of the classical pathways underlying apoptosis. We therefore assessed whether the apoptosis induced by OPD′ was mediated via caspases. The cells were treated with a pan-caspase inhibitor (Z-VAD-FMK) (Chaabane et al., 2013), but this treatment did not significantly affect the survival of cells subjected to OPD′ treatment (**Figure 3A**). This result implied that OPD′ inhibited PC3 growth by inducing apoptosis via a caspase-independent pathway.

RIPK1 is another protein involved in programmed cell death (Luan et al., 2015). RIPK1 is cleaved by cleaved-RIPK1 (C-RIPK1), and also by cleaved-caspase 8 (C-caspase 8). We therefore evaluated the effects of OPD′ on the expression of RIPK1 protein in PC3 cells. Following treatment with several different concentrations of OPD′ (0, 2.5, and 5.0 μM) for 6 h, Western blotting indicated that OPD′ increased the protein expression levels of RIPK1, and decreased the levels of C-RIPK1 and C-caspase 8 (**Figure 3B**). Treatment of cells with OPD′ together with Necrostatin-1 (Nec-1, a RIPK1 inhibitor) reversed the effects of OPD′ on both the FITC^-^/PI^-^ and FITC^+^/PI^-^ areas (**Figure 3C**), without any significant effect on the FITC^+^/PI^+^ and FITC^-^/PI^+^ areas, compared to treatment with OPD′ alone. These findings showed that Nec-1 inhibited the effects of OPD′ on cell viability and early apoptosis, indicating that OPD′ induced RIPK1-dependent cell death.

### OPD′ Increases the RIPK1-Mediated Expression of Bim to Induce Mitochondrial Damage

**FIGURE 4:**
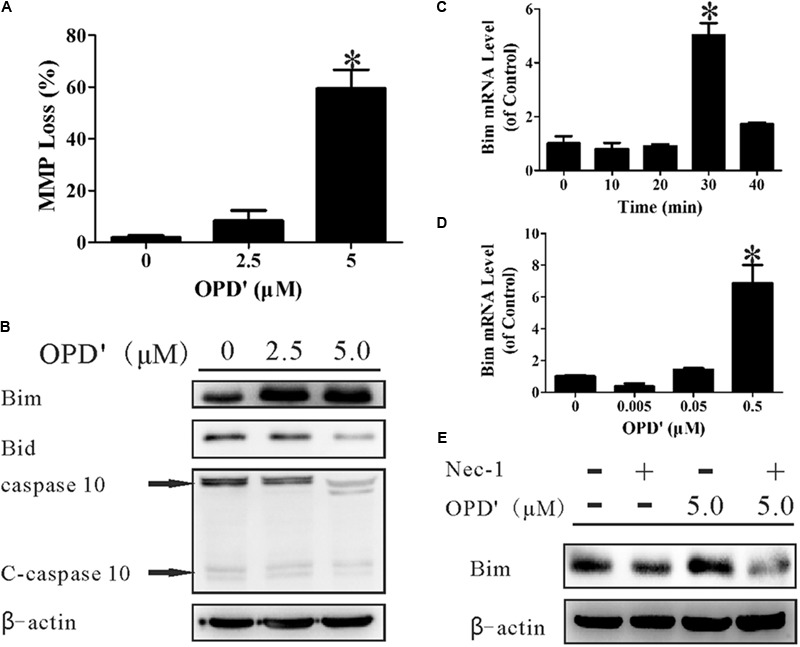
OPD′ increased the expression of Bim and induced mitochondrial membrane depolarization (loss of MMP). **(A)** The MMP loss (%) after treatment with 0, 2.5, or 5.0 μM OPD′ for 6 h. ^∗^*p* < 0.05 vs. the 0 μM group. **(B)** The protein expression levels of Bim, Bid, caspase 10, and C-caspase 10 were examined by Western blotting after treatment of PC3 cells with 0. 2.5, or 5.0 μM OPD′ for 6 h. **(C)** The mRNA expression of Bim was examined by RT-PCR at different time points after treatment with 0.5 μM OPD′. ^∗^*p* < 0.05 vs. the 0 min group. **(D)** After treatment with various concentrations of OPD′ (0.005, 0.05, 0.5 μM) for 30 min, the mRNA expression level of Bim was examined by RT-PCR. The data shown in parts **(A,C,D)** represent the means ± SD of three independent experiments. ^∗^*p* < 0.05 vs. the 0 μM group. **(E)** After pre-treatment with 10 μM Nec-1 for 2 h, PC3 cells were treated with 5 μM OPD′ or the vehicle control for 6 h, then the Bim protein levels were checked by Western blotting. The data shown in parts **(B,E)** represent the means ± SD of three independent experiments. These panels represent one of the replicates.

A decrease in the MMP, which leads to mitochondrial dysfunction, plays an important role in apoptosis (Nakagawa et al., 2005). We therefore examined the effects of OPD′ on the MMP by staining cells with JC-1, an indicator of the MMP. Treatment with 5 μM OPD′ induced a 59.38% decrease in the MMP, compared with a 1.86% decrease in control cells, after 6 h of treatment (**Figure 4A**). Bim and BH3 interacting-domain death agonist (Bid), members of the Bcl2 family, are key proteins involved in the membrane permeability transition (MPT). As illustrated in **Figure 4B**, treatment with OPD′ increased the expression of Bim protein and reduced the levels of Bid and caspase 10. OPD′ treatment also induced Bim mRNA expression by 5.03-fold after 30 min of treatment (**Figure 4C**). Of note, treatment with OPD′ induced the mRNA expression of Bim in a concentration-dependent manner, and treatment with 0.5 μM OPD′ increased the Bim mRNA expression by 6.88-fold (**Figure 4D**). When the cells were treated with the RIPK1 inhibitor, Nec-1, the increase in Bim protein expression induced by OPD′ treatment was prevented (**Figure 4E**). This further supported that the OPD′-induced apoptosis was RIPK1-dependent.

### OPD′ Decreases the Growth of PC3 Xenograft Tumors

**FIGURE 5:**
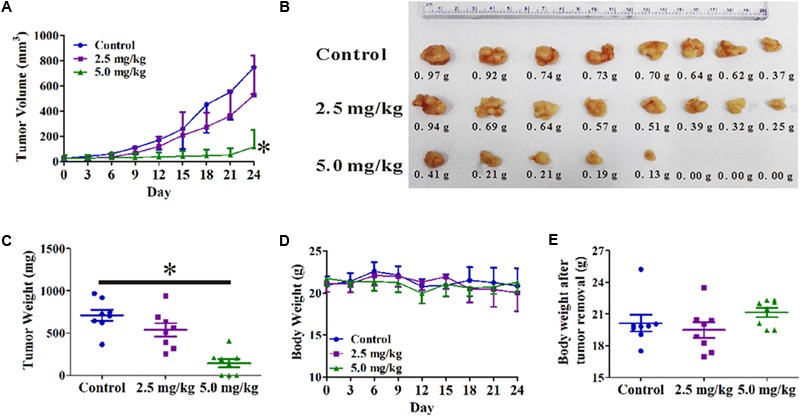
The *in vivo* effects of OPD′ in nude mice bearing PC3 xenograft tumors. OPD′ was injected intraperitoneally (i.p.) at doses of 2.5 and 5.0 mg/kg/d, 5 d/wk for 24 days. Each group comprised eight mice. **(A)** The tumors were measured every 3 days to assess their growth. ^∗^*p* < 0.05 vs. the control group. **(B)** Representative tumors were photographed after removal at the end of the experiment. **(C)** The weight distribution of the tumors at the end of the experiment. ^∗^*p* < 0.05 vs. the control group. **(D)** Animals were monitored for changes in body weight every 3 days as an indicator of toxicity. **(E)** The body weights of the animals after removal of the tumor.

A PC3 xenograft tumor model was established in nude mice to evaluate the anti-tumor activity of OPD′ *in vivo*. As shown in **Figure 5A**, OPD′ treatment led to significant tumor growth inhibition at a dose of 5.0 mg/kg bodyweight, beginning on Day 6 of treatment (*p* = 0.034). At the end of the study (Day 24), the tumor tissues were excised, photographed, and weighed. As shown in **Figures 5B,C**, treatment with 5.0 mg/kg bodyweight OPD′ resulted in significant (*p* = 0.000) tumor growth inhibition by approximately 79.8% on Day 24 compared to the vehicle treatment. OPD′ also resulted in a 24.4% reduction in tumor weight on Day 24 when administered at a dose of 2.5 mg/kg bodyweight, but this decrease was not statistically significant (*p =* 0.078). The 5.0 mg/kg dose led to significantly stronger tumor growth inhibition than the 2.5 mg/kg dose (*p* = 0.000). There was not a significant loss of body weight in any of the groups (**Figures 5D,E**, *p* > 0.05).

### The Growth Inhibitory Activity of OPD′ Is Not Cell Line-Specific

**FIGURE 6:**
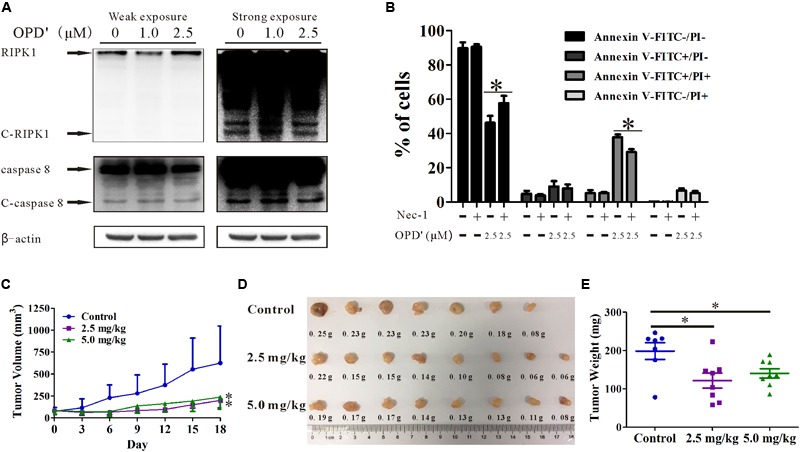
The effects of OPD′ on DU145 prostate cancer cells *in vitro* and *in vivo.* **(A)** DU145 cells were exposed to various concentrations of OPD′ for 6 h, then the protein levels of RIPK1, C-PIPK1, caspase-8, and C-caspase 8 were examined by Western blotting. *n* = 3 independent experiments; this panel represents one of the replicates. **(B)** After pre-treatment with 10 μM Nec-1 for 2 h, the cells were exposed to various concentrations of OPD′ for 18 h, followed by the Annexin V-FITC/PI assay. Data are presented as the means ± SD; *n* = 3 independent experiments. ^∗^*p* < 0.05 vs. 2.5 μM OPD′ alone. **(C)** The tumors were measured every 3 days to assess their growth. OPD′ was injected i.p. at doses of 2.5 and 5.0 mg/kg/d, 5 d/wk for 18 days. Each group comprised eight mice. One of the mice in the control group died on Day 12. ^∗^*p* < 0.05 vs. the control group. **(D)** Representative tumors were weighed and photographed at the end of the experiment. **(E)** The weight distribution of the tumors at the end of the experiment. ^∗^*p* < 0.05 vs. the control group.

In order to confirm the anti-prostate cancer activity of OPD′, we examined the effects of OPD′ on DU145 cells, another androgen-independent prostate cancer cell line. The results showed that exposure to 2.5 μM OPD′ increased the protein expression level of RIPK1 and decreased the levels of C-RIPK1, caspase 8, and C-caspase 8 in DU145 cells, while 1.0 μM OPD′ did not have a significant effect (**Figure 6A**). The compound decreased cell viability (decreased the FITC^-^/PI^-^ area) and induced DU145 cell apoptosis (late apoptosis and necrosis; indicated by the increased FITC^+^/PI^+^area). Nec-1 treatment inhibited this effect of OPD′ (**Figure 6B**). In a DU145 tumor xenograft model, OPD′ induced significant tumor growth inhibition at both 2.5 and 5.0 mg/kg bodyweight, beginning on Day 6 (*p* = 0.002 and 0.002). The weight of the tumor tissues excised on Day 18 were significantly decreased by approximately 40.0 and 30.0% following treatment with 2.5 and 5.0 mg/kg OPD′, respectively (*p* = 0.007 and *p* = 0.035; **Figures 6D,E**). Due to the formation of acini in the DU145 xenograft tumors, the results of the tumor volume measurement (**Figure 6C**) were more pronounced than the results for the tumor tissue weight (**Figure 6E**).

## Discussion

Prostate cancer is the second most common cancer of men and the fifth most common cancer overall worldwide (Torre et al., 2015). Plants have been used as traditional medicine for the treatment of prostate cancer, and many natural products have been isolated from plants and tested for their tumor selectivity and cytotoxic activity. Because of their apparently safety and efficacy, triterpenoid saponins have been gaining increasing interest for cancer therapy (Du et al., 2014; Singh et al., 2017). The anticancer triterpenoid saponins modulate numerous signaling targets related to angiogenesis, apoptosis, autophagy, cancer stem cells, inflammation, metastasis, microRNAs, multidrug resistance, proliferation, and oxidative stress (Liu et al., 2013; Cheng et al., 2014; Chun et al., 2014; Du et al., 2014). Our previous studies have shown that Platycodin D (PD), another triterpenoid saponin, has potent activity against prostate (Zhou et al., 2015) and breast/mammary gland (Kong et al., 2016) cancers.

Natural compounds can induce the apoptosis of cancer cells by triggering caspase and mitochondria-dependent cascades, by inhibiting oncogenes, or by suppressing NFκB signaling (Gioti and Tenta, 2015; Zhou et al., 2015). We herein demonstrated that OPD′ could induce apoptosis in PC3 cells via a caspase-independent pathway. Recent genetic and biochemical evidence has indicated that upregulated signaling by RIPK1 can induce caspase-independent apoptosis. RIP1 was initially discovered as an interaction partner for the first apoptosis signal receptor (Fas) (Stanger et al., 1995). Later, the RIP1 death domain (DD) was reported to be necessary for its binding to other death receptors, such as tumor necrosis factor (TNF)-R1, TRAIL-R1 and TRAIL-R2, and to DD-containing adaptor proteins like TNF-receptor-associated death domain (TRADD) and Fas-associated protein with death domain (FADD) (Stanger et al., 1995; Meylan and Tschopp, 2005). Moreover, RIP1 interacts with a plethora of other adaptor proteins through its intermediate domain (ID), which is also used to recruit other kinases, such as MAP kinase kinase kinase 1 (MEKK1), MEKK3 and RIP3 (Meylan and Tschopp, 2005). In certain situations, RIP1 can also activate mitogen-activated protein kinases (MAPKs), such as p38 MAPK, JNK, and ERK (Festjens et al., 2007). The JNK/c-Jun pathway and its target gene, Fas ligand, are involved in apoptosis (LaRocca et al., 2015). Nec-1, a specific RIPK1 inhibitor, can both block necroptosis and modulate RIPK1-mediated apoptosis. Our present data demonstrated that OPD′ affected RIPK1 and its downstream signaling to induce apoptosis. Watanabe et al. (2017) previously showed that Polyphyllin D, a steroidal saponin derived from *Paris polyphylla*, induced necroptosis via RIPK1 in neuroblastoma cells. Therefore, saponin compounds may exert anti-cancer activity via this newly-discovered RIPK1-mediated (necro) apoptosis pathway.

Several other caspase-independent mechanisms of apoptosis have also been reported. Some of them are associated with the pro-apoptotic actions of non-caspase proteases (e.g., lysosomal proteases and granzymes) (Johnson, 2000), and the proteasomal complex (Ikeda et al., 2009). Others function via apoptogenic mitochondrial proteins like apoptosis-inducing factor (AIF), WOX1, AMID, PRG3, Hspin1, and EndoG (Lorenzo and Susin, 2004). Our present results showed that OPD′ induced PC3 apoptosis via a RIPK1-dependent pathway that led to the up-regulation of the mRNA and protein expression of Bim, which caused mitochondrial membrane depolarization. It was previously reported that avicins, triterpenoid saponins derived from *Acacia victoriae* (Bentham), induce apoptosis via mitochondrial perturbation (Haridas et al., 2001). Kuguaglycoside C, a triterpene glycoside isolated from the leaves of *Momordica charantia*, induces caspase-independent cell death by increasing the expression and cleavage of AIF (Tabata et al., 2012).

It should be noted that the molecular weight of OPD′ is high (>800), indicating that it may be metabolized in the body to generate other potentially bioactive compounds. Previous studies have demonstrated that OPD′ can be decomposed into several glycosides by intestinal microbes (Shen et al., 2005). Diosgenin, one of the metabolites of OPD′, has shown antitumor effects (Jesus et al., 2016). Our present studies showed that OPD′ has direct anticancer effects both *in vitro* and in nude mouse xenograft models. It is possible that the *in vivo* activity might be due to the aglycone or aglucone resulting from the hydrolysis of OPD′ by the microbiome. Our future studies will include an examination of the different metabolites of OPD′, as well as studies to evaluate the anticancer activity of these metabolites.

In addition to the potential limitations associated with investigating only the parent compound *in vitro* in the present study, it should also be kept in mind that our *in vivo* models were developed in mice. Therefore, further studies will be needed to determine whether the results will extrapolate to the human clinical setting.

In summary, the results from our present study indicate that OPD′ has growth inhibitory effects on human prostate cancer cells and xenograft tumors, and these effects are at least partially mediated via its targeting of RIPK1. The current results are promising and provided a basis for further studies of OPD′ as a potential therapeutic agent for androgen-independent/CRPC.

## Ethics Statement

This study was carried out in accordance with the recommendations of laboratory animal care and use of guidelines, Laboratory Animal Welfare and Ethics Committee of the Third Military Medical University. The protocol was approved by the Laboratory Animal Welfare and Ethics committee of the Third Military Medical University. Animal production license (No: SCXK-PLA-20120011). Animal license (No: SYK-PLA-20120031).

## Author Contributions

ZL and HX organized, conceived, designed, and supervised the study. HW, MZ, WS, JW, and YK designed and conducted the experiments and drafted the manuscript. YL, CW, JG, NL, and JL helped in the study design and interpretation of data. All authors read and approved the manuscript.

## Conflict of Interest Statement

The authors declare that the research was conducted in the absence of any commercial or financial relationships that could be construed as a potential conflict of interest.

## Supplementary Material

DATA SHEET S1 Authentication of PC3 cell line.

DATA SHEET S2 Authentication of DU145 cell line.

DATA SHEET S3 Purity test for OPD′.

DATA SHEET S4 Structure test for OPD′.
