# Supplementary figures and images for: Ophiopogonin D′, a Natural Product From Radix Ophiopogonis, Induces in Vitro and in Vivo RIPK1-Dependent and Caspase-Independent Apoptotic Death in Androgen-Independent Human Prostate Cancer Cells
Source: Front Pharmacol. 2018 Apr 30;9:432. doi: 10.3389/fphar.2018.00432 (PMC5936779; doi:10.3389/fphar.2018.00432)

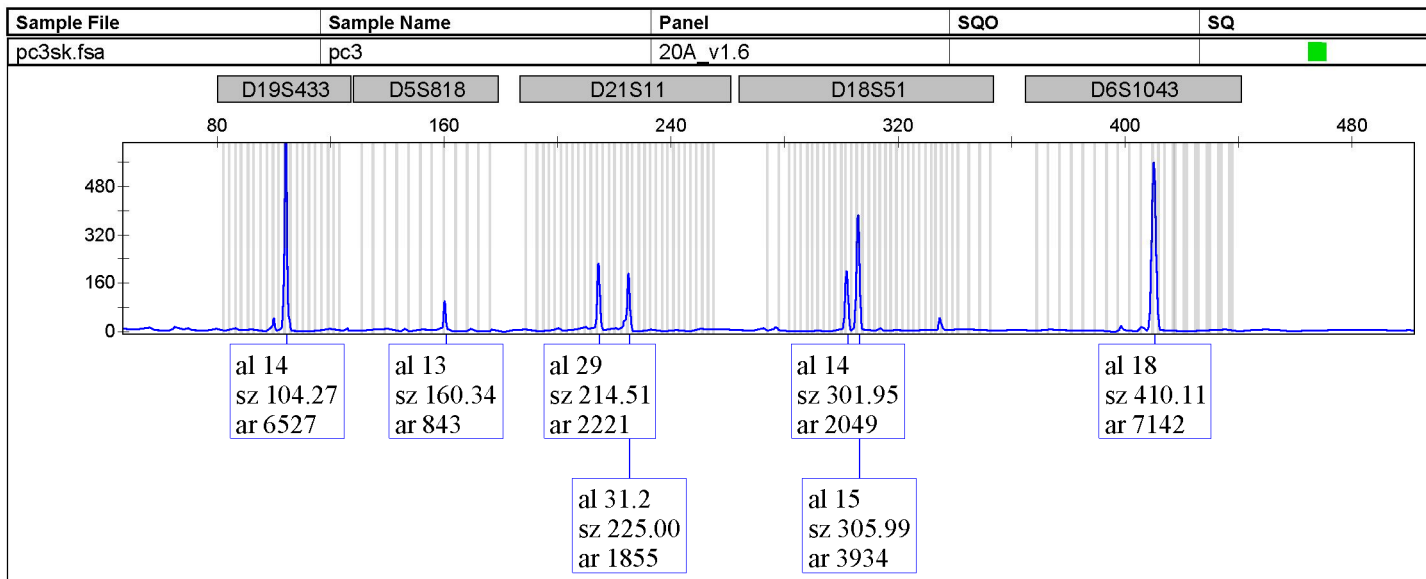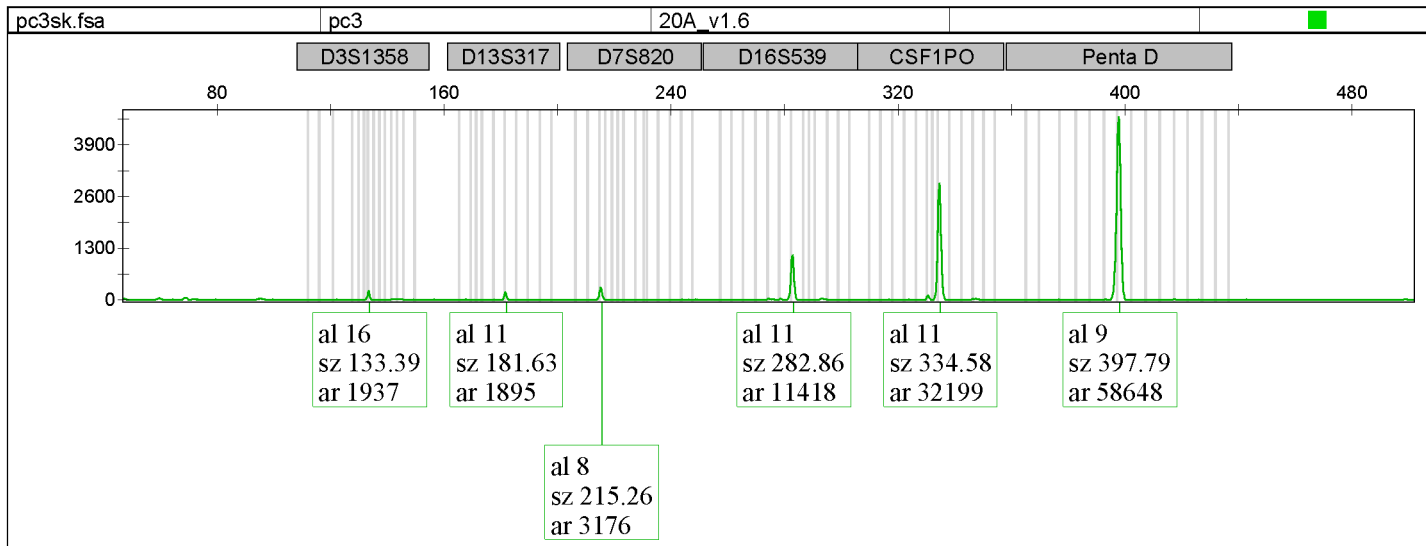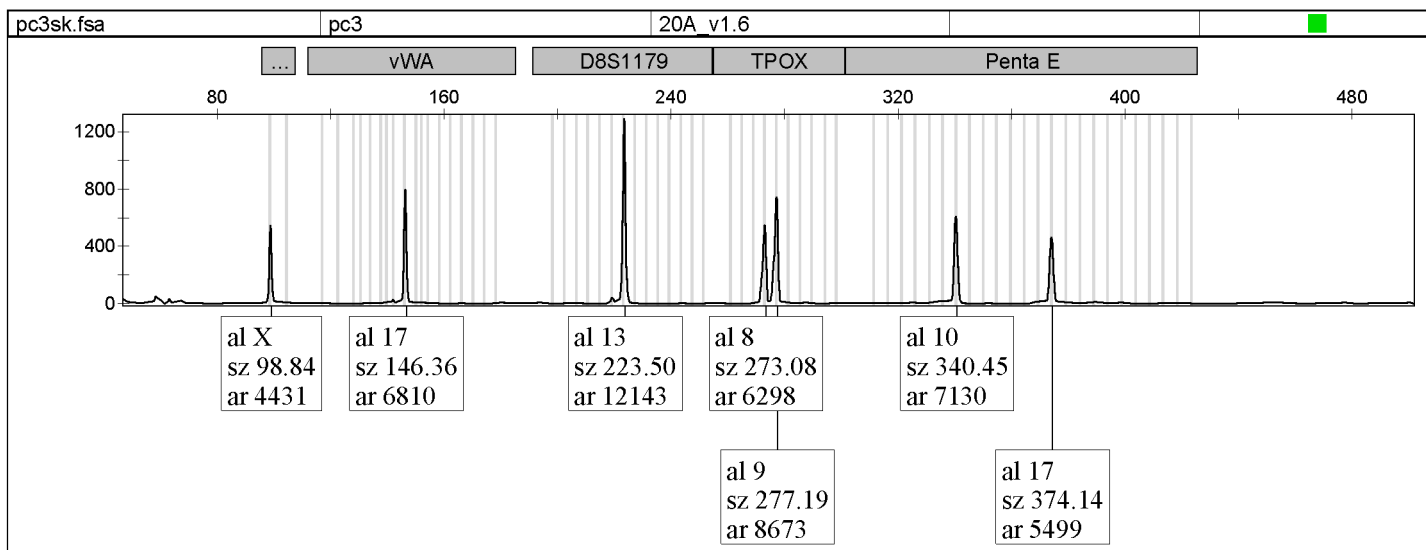

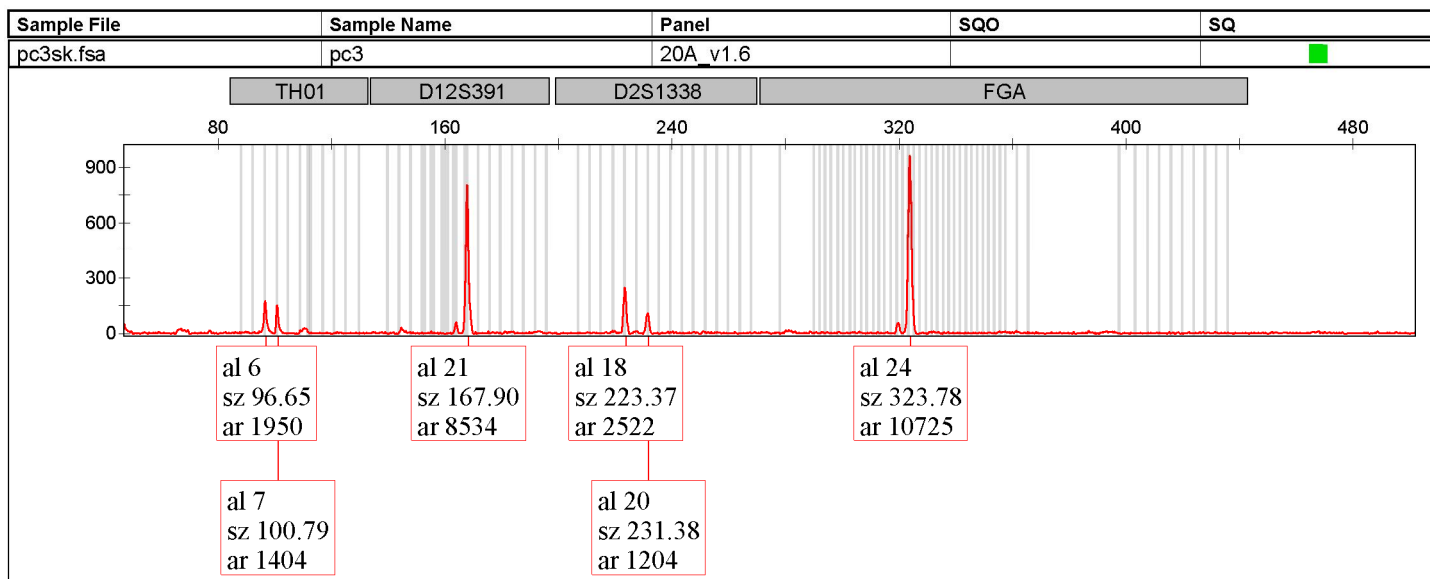

Supplement: DATA SHEET S1 — Authentication of PC3 cell line. [file Data_Sheet_1.PDF]

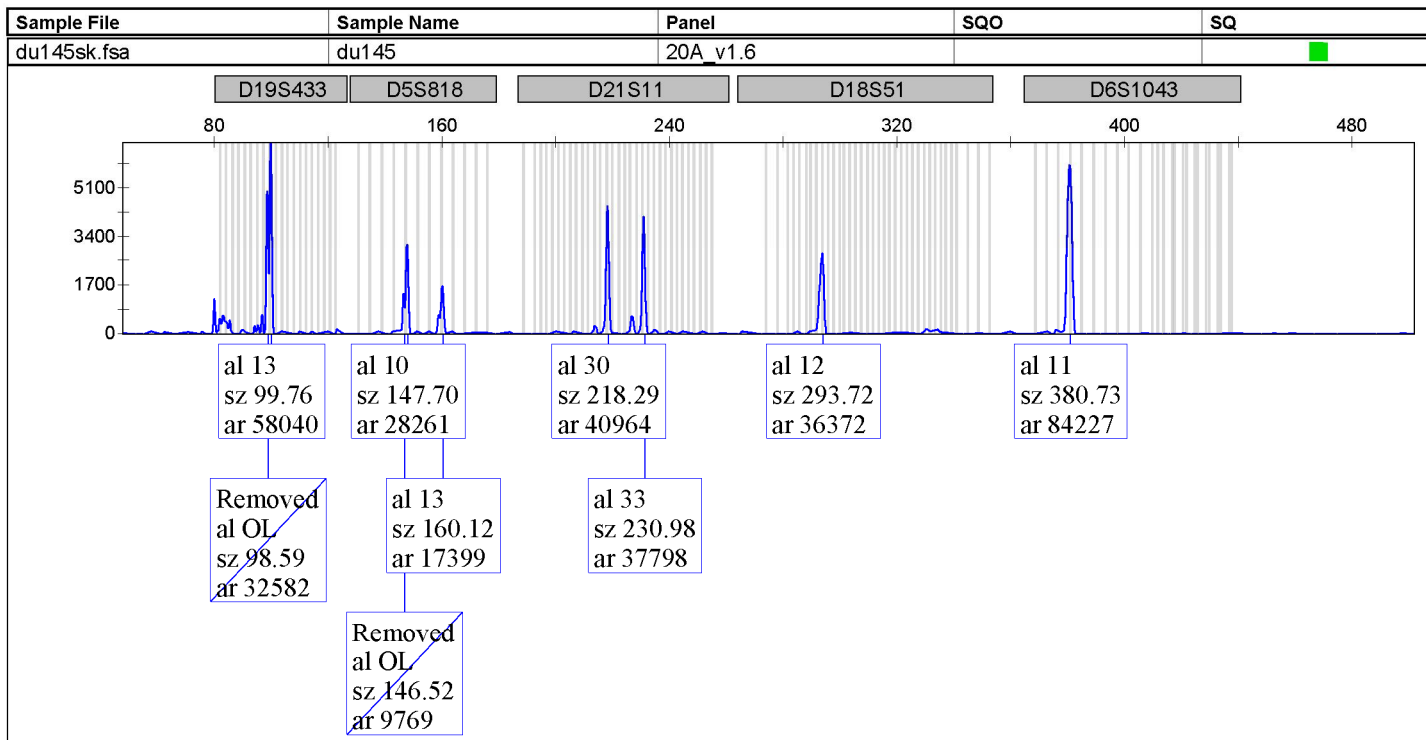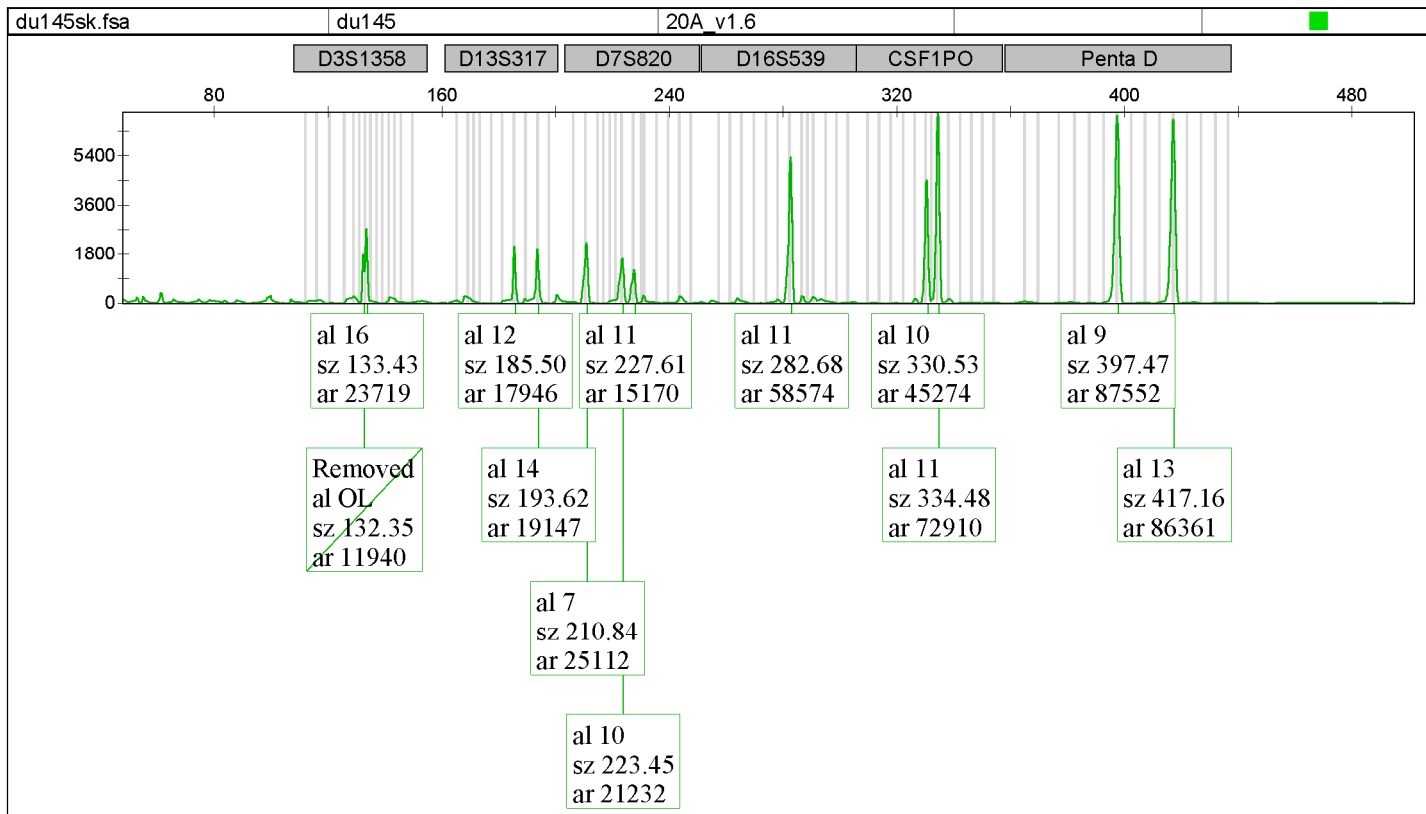

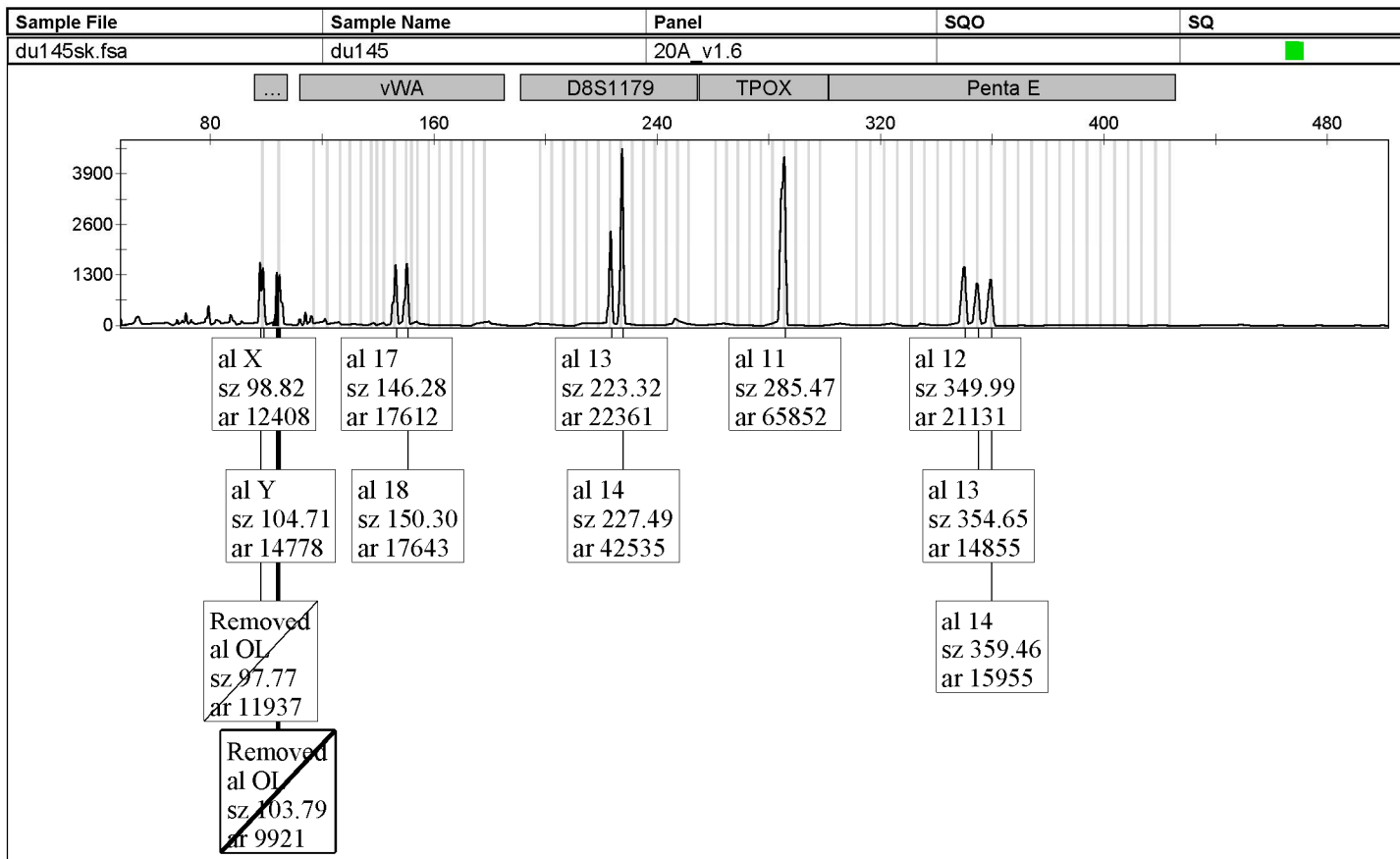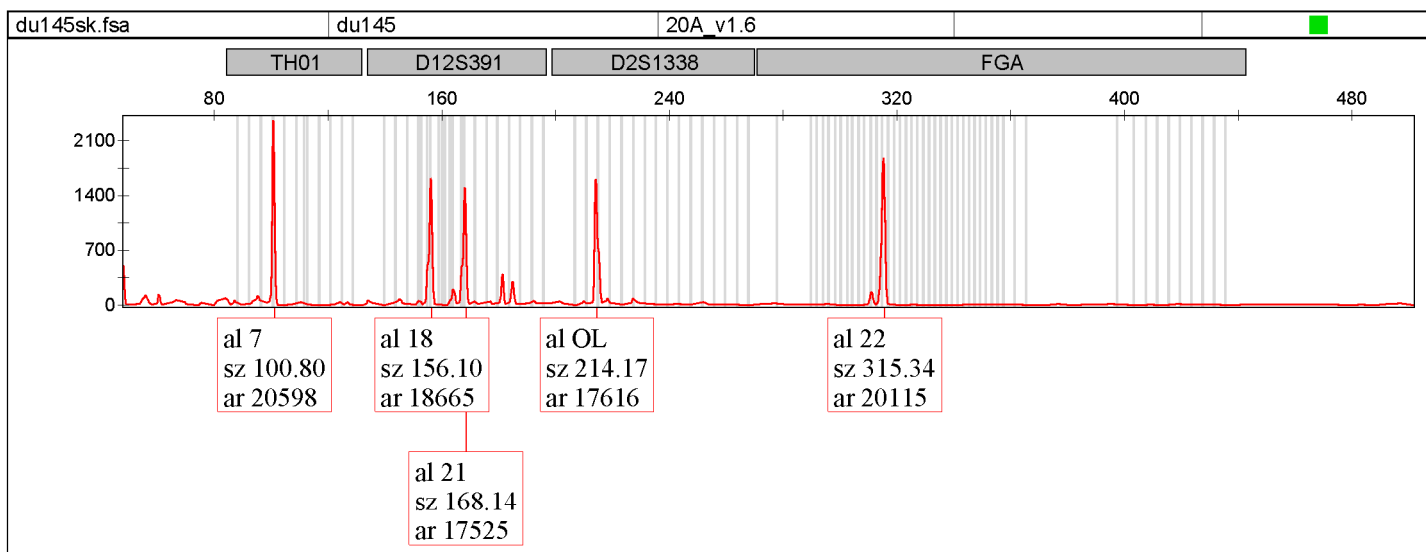

Supplement: DATA SHEET S2 — Authentication of DU145 cell line. [file Data_Sheet_2.PDF]

MD-4

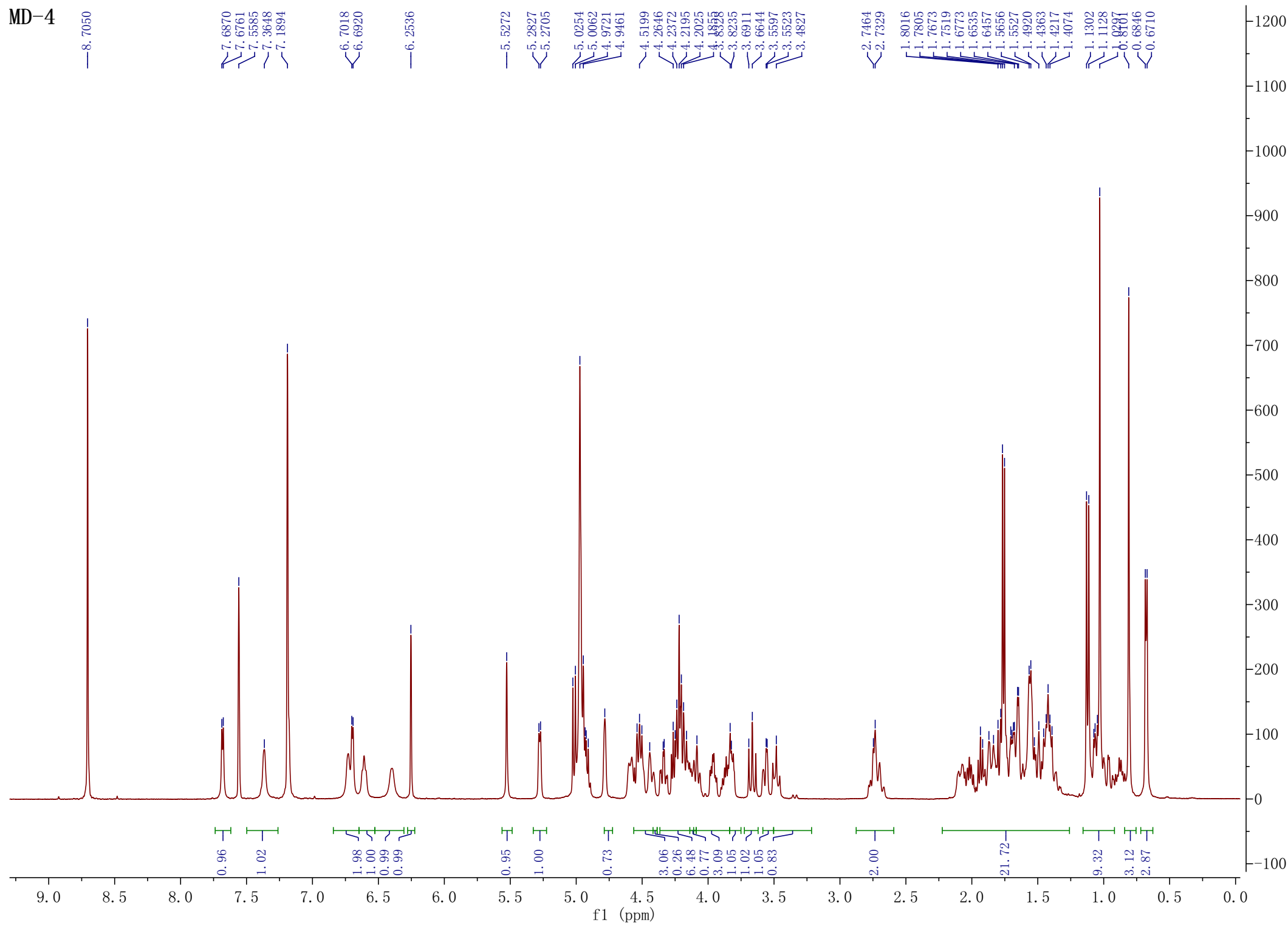

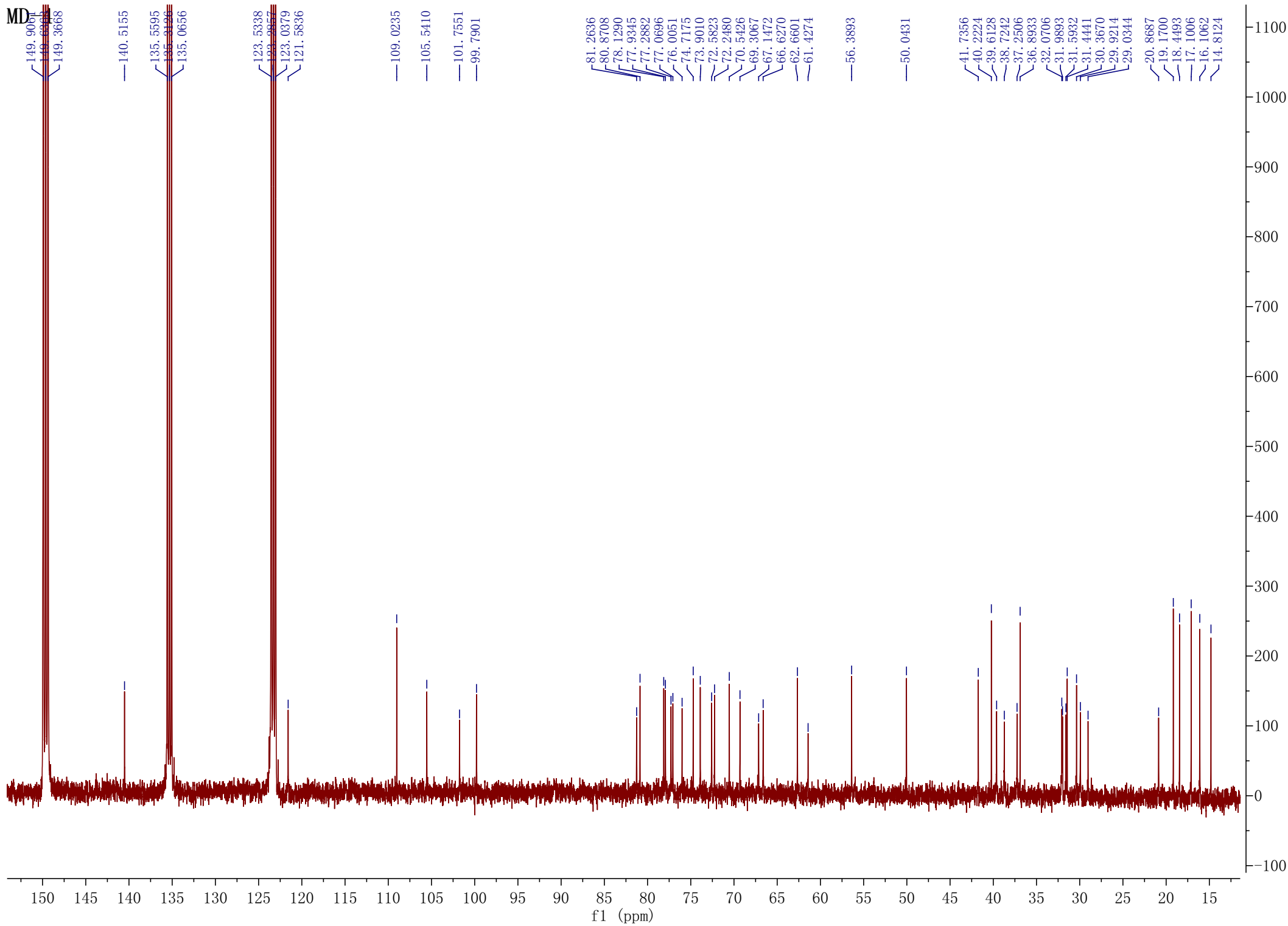

Supplement: DATA SHEET S4 — Structure test for OPD′. [file Data_Sheet_4.PDF]
