# Supplementary material for: Ophiopogonin D′, a Natural Product From Radix Ophiopogonis, Induces in Vitro and in Vivo RIPK1-Dependent and Caspase-Independent Apoptotic Death in Androgen-Independent Human Prostate Cancer Cells
Source: Front Pharmacol. 2018 Apr 30;9:432. doi: 10.3389/fphar.2018.00432 (PMC5936779; doi:10.3389/fphar.2018.00432)
Supplement: DATA SHEET S3 — Purity test for OPD′. [file Data_Sheet_3.PDF]

对照品纯度检测报告

麦冬皂苷 D'

（Ophiopogonin D'）

数据文件:D:\ \麦冬皂苷D`.dat
打印:2017-02-22 15:00:20

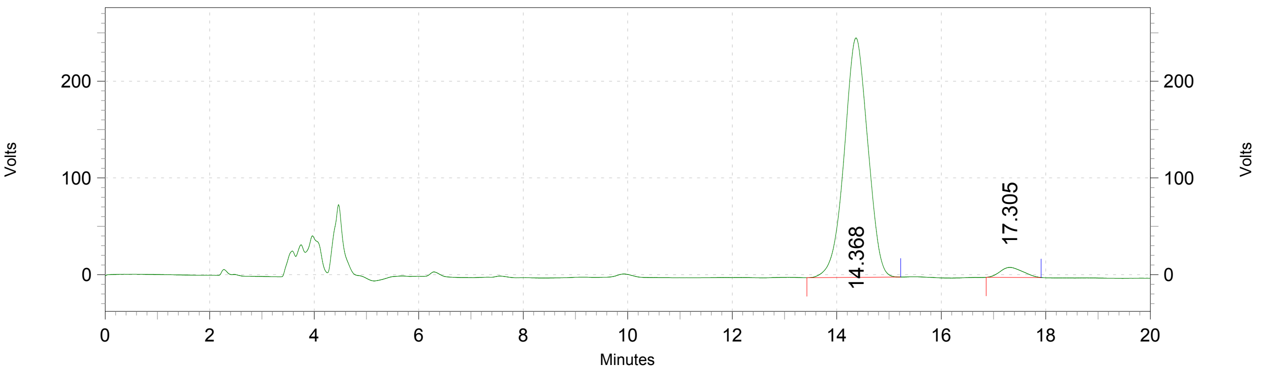

UV1000-203nm

Results

保留时间	面积	面积百分比	峰高	高度百分比
14.368	7738543	96.135	247553	95.991
17.305	311126	3.865	10338	4.009

Totals	8049669	100.000	257891	100.000

流动相方法

	时间（min）	流速(ml/min)	甲醇(%)	水
1	0	0.8	86	14
2	20	0.8	86	14

色谱柱：SinoChrom ODS-BP 5μm 柱温：30℃
